# COVID-19 Anosmia: High Prevalence, Plural Neuropathogenic Mechanisms, and Scarce Neurotropism of SARS-CoV-2?

**DOI:** 10.3390/v13112225

**Published:** 2021-11-04

**Authors:** Fengyi Liang, De Yun Wang

**Affiliations:** 1 Healthy Longevity Translational Research Program, Department of Anatomy, Yong Loo Lin School of Medicine, National University of Singapore, Singapore 117594, Singapore; 2 Infectious Diseases Translational Research Program, Department of Otolaryngology, Yong Loo Lin School of Medicine, National University of Singapore, Singapore 119228, Singapore; entwdy@nus.edu.sg

**Keywords:** COVID-19, SARS-CoV-2, olfactory dysfunction, anosmia, pathogenesis

## Abstract

Severe acute respiratory syndrome coronavirus 2 (SARS-CoV-2) is the causative pathogen of coronavirus disease 2019 (COVID-19). It is known as a respiratory virus, but SARS-CoV-2 appears equally, or even more, infectious for the olfactory epithelium (OE) than for the respiratory epithelium in the nasal cavity. In light of the small area of the OE relative to the respiratory epithelium, the high prevalence of olfactory dysfunctions (ODs) in COVID-19 has been bewildering and has attracted much attention. This review aims to first examine the cytological and molecular biological characteristics of the OE, especially the microvillous apical surfaces of sustentacular cells and the abundant SARS-CoV-2 receptor molecules thereof, that may underlie the high susceptibility of this neuroepithelium to SARS-CoV-2 infection and damages. The possibility of SARS-CoV-2 neurotropism, or the lack of it, is then analyzed with regard to the expression of the receptor (angiotensin-converting enzyme 2) or priming protease (transmembrane serine protease 2), and cellular targets of infection. Neuropathology of COVID-19 in the OE, olfactory bulb, and other related neural structures are also reviewed. Toward the end, we present our perspectives regarding possible mechanisms of SARS-CoV-2 neuropathogenesis and ODs, in the absence of substantial viral infection of neurons. Plausible causes for persistent ODs in some COVID-19 convalescents are also examined.

## 1. Introduction

Apart from causing respiratory, cardiovascular, and systemic problems, COVID-19 is also accompanied by frequent neurological manifestations such as headache, dizziness, anosmia, ageusia, or even stroke [1,2,3,4,5]. Among others, COVID-19-related olfactory dysfunctions (ODs), as represented by anosmia or hyposmia, are highly relevant to upper respiratory infection, as these dysfunctions imply infection and pathology of the olfactory epithelium (OE) lining the superior recess of the nasal cavity. It is thus not surprising that COVID-19 related ODs have attracted much attention from both the clinical and basic medicine research communities [6,7,8,9,10].

ODs are quite common in disorders of the nose. The causes of ODs vary from nasal congestion, OE inflammation, infection or damage, or structural functional abnormalities of the olfactory nerve, olfactory bulb (OB), or other central nervous system (CNS) structures. However, the ODs in COVID-19 seem somehow special in that these deficits are unusually prevalent, sometimes appear before other symptoms, and, on occasions, might even be the only symptoms of severe acute respiratory syndrome coronavirus 2 (SARS-CoV-2) infection without apparent nasal congestion or inflammation. The incidence of smell and/or taste impairment in COVID-19 patients varied from as low as 5% to as high as 98% in the literature, depending on areas, populations, SARS-CoV-2 variants, and methods of diagnosis, but most analyses have reported an OD rate of 20–80% [11,12,13,14,15,16,17,18,19,20,21]. Although a majority of COVID-19-related ODs disappear in a few weeks, the deficits in some patients could persist long after resolution of other COVID-19 abnormalities [22,23,24,25].

The questions then arise as to the reasons for the unusually high prevalence of ODs in COVID-19, the possibilities of SARS-CoV-2 invasion or infliction of acute and chronic damages to the peripheral or central olfactory system, and the prospects of olfaction recovery in the cases of protracted post-COVID-19 ODs. There have been many reports, experiments, or speculations with regard to COVID-19-related ODs within the short time period since the outbreak of the COVID-19 pandemic, one often contradicting the other. Here, we attempt to first discuss possible molecular and cytological substrates for high susceptibility of the OE to SARS-CoV-2 infection. SARS-CoV-2 neurotropism (or the lack of it) and COVID-19 neuropathology will then be analyzed. In view of the scarce neurotropism of the virus, plausible mechanisms of COVID-19 neuropathogenesis and ODs are explored, such as neural support deprivation, inflammation, immune reactions at the OE, anterograde degeneration or molecular trafficking along nerve fibers, and microvascular thrombosis in the OB or other CNS regions. Possible causes of protracted ODs after COVID-19 are also briefly reviewed.

## 2. Cytological and Molecular Basis for High Prevalence of Olfactory Dysfunctions in COVID-19

The sense of smell (olfaction) starts from the binding of airborne odor molecules (odorants) to their receptors on the surface of the OE at the superior part of the nasal cavity. Here, chemical characteristics of the odorants are encoded into electrical signals, and then transmitted monosynaptically through the olfactory nerves (cranial nerves I) to the OB. After relay and integration there, the olfactory impulses are further transmitted to higher order olfactory regions of the CNS for olfactory perception, reactions, memory, and other neural processes [26,27].

### 2.1. Basic Histology and Cytology of the OE

The OE lines the superior vault of the nasal cavity. Its location near the entrance of the upper respiratory tract facilitates early detection of important or potentially harmful odorants in the inhaled air, but this frontline positioning of the special sense receptor organ also renders the OE vulnerable to pathogens or damages in the upper respiratory tract [28,29]. Histologically, the OE is a layer of pseudostratified columnar epithelium, as is the respiratory epithelium (RE) lining most other parts of the nasal cavities and paranasal sinuses. At the cytological level, however, the OE and RE differ significantly from each other. Specifically, the OE is made of ciliated olfactory receptor neurons (ORNs), sustentacular supporting cells, globose and horizontal basal cells, occasional microvillar cells and ductal cells of Bowman’s glands, plus glandular cells of Bowman’s glands in the lamina propria of the olfactory mucosa [28,30,31]. The sustentacular and microvillar cell nuclei usually occupy a more apical position of the OE; ORN cell bodies are mostly located in the middle layer, whereas basal cells are found next to or close to the basement membrane. The nasal RE, however, is a ciliated pseudostratified columnar epithelium made of ciliated and non-ciliated columnar epithelial cells, secretory goblet cells, basal cells, occasional brush cells, small granule cells, and ductal cells of glands, plus glandular cells in the lamina propria [32].

The bipolar ORNs are directly exposed, at the dendritic knob and cilia, to the nasal mucus and nasal cavity environment. While the direct interaction with the inhaled air enables a high sensitivity to odorants in the immediate environment, the direct contact with nasal mucus and air subjects the ORNs to the risk of potential harm by detrimental molecules or microorganisms that are breathed in and out of the nasal cavity. Probably because of this vulnerability, the ORNs have a relatively short lifespan of only a few weeks and are constantly replaced by new receptor neurons generated from OE basal cells [28,33].

At the axonal end, the ORNs are monosynaptically connected with neurons of the olfactory bulb of the CNS [27,28]. The olfactory nerve not only conducts olfactory nerve impulses to the olfactory bulb but may also serve as a trafficking pathway for certain intrinsic or extrinsic molecules, toxins, or viruses along the axoplasm from the OE to the OB, or vice versa. As compared with trafficking through the blood stream and blood–brain barrier, the olfactory nerve represents an alternative and more direct route of CNS vulnerability to infections/toxicities of nasal origin [34,35,36,37]. The direct neural pathway and its trafficking capability are sometimes also used for delivering therapeutics or other molecules to the CNS, to bypass the blood–brain barrier [38,39,40,41,42].

### 2.2. Why Is the OE Especially Susceptible to SARS-CoV-2 Infection?

In terms of luminal surface area, the OE accounts for only about 5% of the total nasal epithelium in humans [7,43,44], but ODs (anosmia, hyposmia, etc.) have been reported in up to about 80% of COVID-19 patients, and ODs are sometimes the first or only clinical manifestation of the infection [11,12,13,14,15,16,17,18,19,20,21]. Sudden anosmia has been reported to be even more predictive of SARS-CoV-2 infection than any other symptoms, including fever, cough, hoarse voice, or shortness of breath [45].

**Figure 1:**
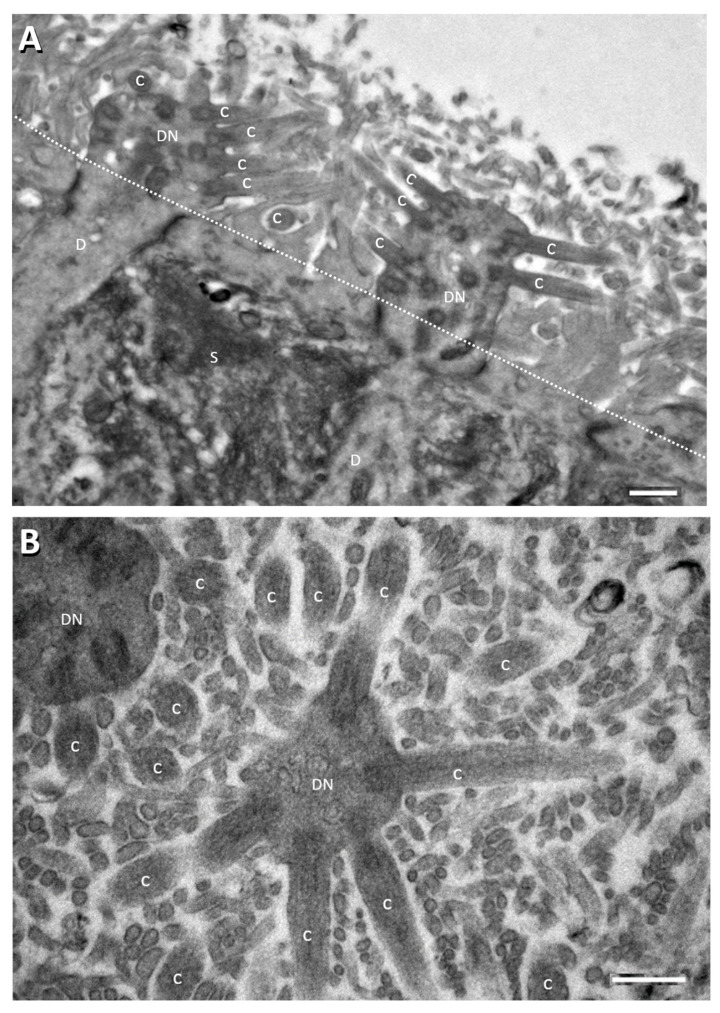
Electron micrographs showing perpendicular (**A**) and tangential/oblique section (**B**) of the apical part of the rat OE. Dotted line in panel A denotes sustentacular cell (S) apical surface from which the long thin sustentacular-cell microvilli protrude into the nasal cavity for about 2–3 µm. ORN dendritic knobs (DN) and cilia (C) at apical ends of ORN dendrites (D) are mostly found among the sustentacular microvilli (most of the unlabeled small profile structures in (**B**) and in area above the dotted line in (**A**). Human OE is similarly organized [52,53,54]. Scale bars = 0.5 µm.

The disproportionately high prevalence and specificity of ODs suggest high susceptibility of the OE to SARS-CoV-2 infection. Why is this so? There is no definitive answer to the question yet, but difference in expression of angiotensin-converting enzyme 2 (ACE2, the SARS-CoV-2 receptor) has been well noted between the OE and RE. There have been reports of more abundant ACE2 expression in the OE (up to hundreds of times more in immunofluorescence intensity, as quantified by laser scanning confocal microscopy) than in the neighboring nasal RE [46,47,48] (see below for further details concerning ACE2 expression in specific cell types of the OE, RE, and some other tissues). Besides, structurally, the OE luminal surface is mostly occupied by thin and long microvilli that are rooted from the apical surface of olfactory sustentacular cells. This coat of microvilli could effectively increase dozens-fold to hundred-fold the apical surface area of OE sustentacular cells (Figure 1). In contrast, few cells of the nasal RE bear apical microvilli. Even though the motile apical cilia of respiratory epithelial cells could also multiply the surface area, this cilia mechanism might not effectively serve the purpose for increased viral binding. Coordinated cilia motility actually propels out pathogens, particles, and cell debris to clean up the airway [49,50]. Cellular microvilli, in contrast, are well known for functional roles to increase cellular surface area for binding or absorption [51]. The possibility of OE sustentacular cell microvilli as an effective areal multiplier for binding SARS-CoV-2 is further supported by the presence here of ACE2 receptor for the virus (see below), although it awaits future experimental evidence to verify this notion specifically.

## 3. Neurotropism and Neuropathology of SARS-CoV-2?

Various molecules or viruses can preferentially bind to and enter neuronal cells, migrate along neuronal axoplasm, and are thus neurotropic. Some neurotropic molecules are of intrinsic origins from the nervous system or other parts of the body (such as NGF, BDNF, and NT-3) [37]. Other neurotropic molecules are of extrinsic origins, including bacterial or fungal toxins (such as cholera toxin, tetanus toxin, and botulinum neurotoxin) and lectins (such as wheat germ agglutinin (WGA) and phaseolus vulgaris leucoagglutinin (PhA-L)) [35,55,56,57,58]. A number of viruses, including the herpes simplex viruses, poliovirus, rabies virus, and bovine herpesvirus 5 (BHV5), are also neurotropic [34,36,59,60,61]. A majority of these neurotropic molecules/viruses have designated binding receptors identified on neuronal cells, and thus could enter neurons through receptor-mediated endocytosis. Many of them can also be transported anterogradely or retrogradely along the neuronal processes (mostly axons) or neural pathways. A few are even capable of travelling transsynaptically (transneuronally) from one neuron to other neurons or effector cells through synaptic connections. The anterograde, retrograde, and transsynaptic trafficking properties of some neurotropic molecules (such as WGA, PHA-L, cholera toxin) or viruses (such as HSV) have been exploited for tracing neuronal connections in the nervous system [58,62,63,64,65].

### 3.1. SARS-CoV-2 Receptor and Uptake Priming Protein in the OE and Related Structures

A few groups examined the expression of ACE2 (the SARS-CoV-2 receptor) and transmembrane serine protease 2 (TMPRSS2, the SARS-CoV-2 cell entry-priming protease) to assess possible tropism of the virus to specific tissues and cell types [66,67]. To that end, approaches such as single-cell RNA-sequencing, immunocytochemistry, immunohistochemistry, and in situ hybridization histochemistry have been employed. The results still somehow vary, but basic expression patterns in the OE, RE, and elsewhere have emerged. In both human and mouse OE, ACE2 immunoreactivity has been found mainly on the sustentacular cells, especially at the supranuclear part, apical surface, and microvilli of sustentacular cells. To a variable extent, ACE2 could also be seen on OE basal cells, ductal and glandular cells of Bowman’s glands. Minimal or no ACE2 was detected on mature ORNs. TMPRSS2 exhibited a cell-type expression pattern similar to ACE2, albeit less cell type-specific [46,47,48,68]. In human nasal RE, ACE2 has been detected in ciliated columnar respiratory epithelial cells, but not in the secretory goblet cells [69]. Another study, in contrast, found ACE2 on RE secretory cells [47].

In the CNS, including the olfactory bulb, most studies have observed significant ACE2 in brain vasculature, but no or little ACE2 in neurons or glia [68,70,71]. ACE2 was found on vascular endothelial cells and on pericytes of capillaries. TMPRSS2 transcripts were barely detected in the mouse CNS [46]. One group, however, reported expression of ACE2 and TMPRSS2 in astrocytes and microglia, in addition to brain vascular endothelial cells and pericytes [72].

### 3.2. SARS-CoV-2 Infection and COVID-19 Pathology in the OE and Related Structures

Many groups have examined SARS-CoV-2 infection and/or pathology in various cells, tissues, and organ systems in human autopsy or biopsy samples, and in infected model animals such as the nonhuman primates, golden Syrian hamster, ferret, and hACE2 transgenic mouse. Here, we briefly examine the data related to the nervous system, especially the OE and OB. In COVID-19 human autopsy, SARS-CoV-2 spike protein was detected in many OE sustentacular cells and RE epithelial cells, and occasionally in probable progenitor ORNs positive for Olig2 [73]. Olfactory mucosa showed the highest SARS-CoV-2 viral load as compared to the oral mucosa, cornea, conjunctiva, olfactory bulb, olfactory tubercle, trigeminal ganglion, medulla oblongata, and cerebellum [74]. Another recent study identified SARS-CoV-2 RNA in brush biopsy from the OE of COVID-19 patients. SARS-CoV-2 nucleoprotein was found mainly in sustentacular cells and immune cells. Occasional ORN infections were seen, but the number seemed insignificant compared to the uninfected control group [23]. In terms of pathology, human COVID-19 autopsies revealed focal atrophy, white blood cell infiltration in the olfactory mucosa, ORN axon damage, and inflammatory neuropathy of the olfactory nerve [74,75]. Strong cleaved caspase-3 signals indicative of cell apoptosis have also been observed in both infected and noninfected cells in the olfactory mucosa of anosmic COVID-19 patients [23]. Biopsy in a case of persistent anosmia 3 months after COVID-19 infection found massive disruption and desquamation of olfactory epithelium [22].

In golden Syrian hamster, massive OE damage and immune reactions were seen after nasal instillation of SARS-CoV-2, resulting in desquamation of the OE and infiltration of immune cells. A large number of sustentacular cells were infected. ORNs showed loss of cilia, but little or no evidence of viral infection [76,77,78,79]. In other studies, infection of some basal cells and occasional ORNs could not be ruled out [23,77]. In keeping with the findings from human autopsy and golden Syrian hamster model, SARS-CoV-2-infected ferrets also showed virus positivity in OE sustentacular cells and nasal respiratory epithelial cells [80].

Possible infection and pathology of other PNS structures in COVID-19 remain largely unexplored. The pathology of ageusia, for example, seems mostly unknown, although viral load in saliva has been found correlated with COVID-19 symptoms and loss of taste [81]. ACE2 was low or absent in taste receptor cells [7,82]. Noticeably, however, SARS-CoV-2 nucleoprotein has been detected in subsets of autopsy cranial nerves originating from the lower brainstem [83]. Diffused low ACE2 expression has been reported in some dorsal root ganglion neurons [84]. SARS-CoV-2 was rarely detected in the cerebrospinal fluid (CSF) of COVID-19 patients, including those with severe neurological manifestations or complications, but inflammatory or immunoreactive alterations seem common [9,85,86,87,88,89,90].

Regarding the CNS, brain autopsies in COVID-19 have generally revealed no SARS-CoV-2 in neurons or glia. PCR assays had found viral RNA in some brain tissue lysates, probably from virions or viral RNAs in infected blood or blood vessels of the brain [3,91,92,93]. Indeed, SARS-CoV-2 spike protein was detected in vascular endothelial cells and in microthrombi in COVID-19 brain autopsy samples including the OB, cerebellum, and brainstem [74]. Another autopsy study revealed occasional presence of viral N- or S-protein in individual cells of unknown identity in the CNS but found no direct relation of the cellular infection to major CNS pathological changes [83]. Pathological findings from COVID-19 autopsies include extensive inflammation, microglia activation, astrogliosis (especially in OB and medulla oblongata), perivascular infiltration of cytotoxic T lymphocytes or leukocytes, intravascular microthrombi [74,75,83,92], and hypoxia-associated alterations [93]. Brain imaging abnormalities, indicative of edema, injury, and microbleeding, have also been reported in the olfactory bulb of COVID-19 patients [94,95,96].

In experimental animals, irrespective of SARS-CoV-2 infection of the RE and OE, there has been no report of substantial invasion of the virus into the CNS neurons or glia (including the OB) [10,76,77,78,79,80,97,98,99], with a few exceptions (see below). SARS-CoV-2 nucleoprotein-positive myeloid cells were occasionally observed in the OB, but the exact identity (blood monocytes, macrophages, or CNS microglia) and locations (intravascular or extravascular) of these cells remained uncertain [23]. Likewise, although mostly undetectable in neurons or glia in the brain (including the OB), SARS-CoV-2 could sometimes be recovered from brain samples of infected animals, probably from infected blood or vascular endothelial cells [23,78]. Neuropathological alterations after SARS-CoV-2 infection of susceptible experimental animals ranged from absence of clear changes to inflammation, microglia activation, and infiltration of macrophages, similar to autopsy findings in human COVID-19 [76,77]. One exception is the K18-hACE2 transgenic mice that overexpress human ACE2 transgene (hACE2) under human K-18 promotor control and display unusually high sensitivity to SARS-CoV-2. Intranasal infection of K18-hACE2 transgenic mice could result in not only viral invasion of the OE, RE, and lungs, but also extensive virus spread into CNS regions such as the OB, anterior olfactory nucleus, thalamus, hypothalamus, and cerebral cortices [100,101]. In contrast, another line of transgenic mice that overexpresses hACE2 under the mouse ACE2 promotor control also suffers from SARS-CoV-2 infection and disease but did not show prominent virus spread to the CNS [102]. Even though seemingly unrepresentative, the K18-hACE2 transgenic mouse model appears suitable for therapeutic screening, as evidenced by the effectiveness of COVID-19 convalescent antisera in preventing disease or mortality by SARS-CoV-2 in these mice [101].

## 4. Olfactory Neuropathogenesis in COVID-19

### 4.1. Pathogenesis in the OE upon SARS-CoV-2 Infection

**Figure 2:**
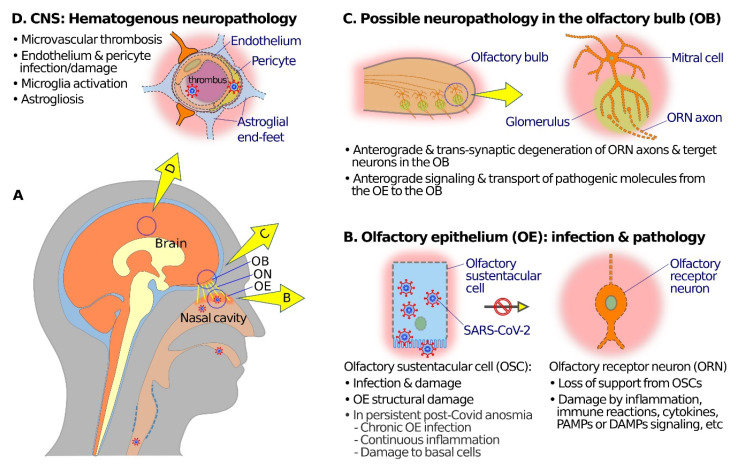
Schematic diagrams showing possible mechanisms of olfactory neuropathogenesis in COVID-19. (**A**) A schematic overview to illustrate relations among nasal cavity, olfactory epithelium (OE), olfactory nerve (ON), olfactory bulb (OB), and the brain. (**B**) At the OE, SARS-CoV-2 mainly infects olfactory sustentacular cells (OSCs) that express high levels of SARS-CoV-2 receptor ACE2 on the luminal surface. Sustentacular cell infection and damage may lead to inflammation, immune reactions, release of cytokines, and signaling through pathogen-associated molecular patterns (PAMPs), damage-associated molecular patterns (DAMPs), and pattern recognition receptors (PRRs) which in turn may cause dysfunctions (such as anosmia or hyposmia) and damage and/or anterograde degeneration of olfactory receptor neuronal cells (ORNs). In the case of post-COVID-19 persistent olfactory dysfunctions, pathogenic mechanisms may include damage of basal cells, continuous inflammation, or chronic SARS-CoV-2 infection in the OE. (**C**) Anterograde degeneration, signaling, and transport of pathogenic molecules from the OE to the OB along ORN axons may result in dysfunction and transsynaptic degeneration of neural structures in the OB. (**D**) SARS-CoV-2 infection of endothelial cells or pericytes, and microthrombi in capillary blood vessels, may compromise the blood–brain barrier, and give rise to hematogenous neuropathology and dysfunctions in various brain regions, including the OB.

In summary, SARS-CoV-2 at the OE mainly infects the olfactory sustentacular cells (Figure 2A,B). Although OE horizontal basal cells have been shown to express moderate ACE2, these cells are normally not exposed to the nasal cavity and mucus, and thus might contract the virus only under special circumstances, such as compromise in OE integrity or secondary infection. OE desquamation, disruption, cell death, ORN cilia loss, and other pathological changes after SARS-CoV-2 infection, however, affect both ORNs and non-neuronal OE cells. Then, how are the mainly sustentacular cell infection and damages translated into ORN dysfunctions and OE pathology?

First, SARS-CoV-2-elicited sustentacular cell damages or death would compromise OE structural integrity, and significantly deprive the ORNs of the usual supports from non-neuronal, especially sustentacular, cells for structural stability, metabolism, homeostasis, and olfactory functions. The loss of supports might cause ORN injuries or even cell deaths. In case of infection and destruction of Bowman’s glands or ducts, OE mucus secretion would be adversely affected, and possible infection of OE basal cells or precursor ORNs may also hinder regeneration and functional recovery of the OE [31,103,104,105,106,107,108].

More importantly, infection of the OE would presumably mobilize immune reactions and activate inflammation as well as the release of specific cytokines or chemokines at the olfactory mucosa that could variably affect ORNs and other OE cells structurally or functionally. OE sustentacular cells are also phagocytic [105]. OE microvillar cells expressing transient receptor potential channel TRPM5 might have a role in neuroimmune detection or reactions [109]. A recent study has further demonstrated an ORN-mediated TrkA-dependent ultrarapid immune response to intranasal viral infection and OE damage in the rainbow trout [110]. Selective upregulation of interferon in the OE inhibits ORN odorant receptor protein expression and induces anosmia even without overt damage to the OE [111]. OE biopsy of COVID-19 patients showed significant increase in tumor necrosis factor alpha (TNF-α) but not IL-1β, as compared to levels in uninfected controls [112]. Transgenic overexpression of TNF-α is known to promote ORN cell death [6,113]. Interleukin 17c (IL17c) and its receptor are present in the mouse olfactory mucosa, and the former is markedly upregulated upon poly I:C intranasal instillation, mimicking viral infection [114].

Based on previous studies, it is also likely that pattern recognition receptors (PRRs) and related damage-associated molecular patterns (DAMPs) or pathogen-associated molecular patterns (PAMPs) play important roles in pathogenesis of SARS-CoV-2 in the OE and RE. PAMPs and DAMPs are involved in epithelial innate immunity and in pathogenesis of many acute and chronic inflammatory diseases. The single-pass transmembranous Toll-like receptors (TLRs), a type of PRRs, for example, recognize specific PAMPs, play important roles in innate immune reactions, and are expressed by neurons and glia of both the CNS and PNS [115]. TLR3, which detects double-stranded RNAs and activates NF-κB, has been shown to be preferentially expressed in mouse OE sustentacular cells [116]. Intranasal infusion of PAMPs and related mimetic molecules to activate TLRs would evoke neuroimmune or inflammatory responses [6,117,118], or protection of the OE from subsequent infection and the CNS from virus invasion [119]. It awaits future investigations to elucidate the involvement details of PRRs, PAMPs, and DAMPs in COVID-19-related olfactory dysfunctions and neuropathology.

In COVID-19 cases with obvious nasal congestion and rhinitis, obstructed airflow through the nasal cavity would also adversely affect olfaction and exacerbate ODs.

### 4.2. Pathogenesis in the OB and Other CNS Structures

The microglia activation, astrogliosis, inflammation, and immune reactions in the OB and related CNS regions after OE SARS-CoV-2 infection appear mostly elicited indirectly, rather than by the invasion of the virus itself [6,10]. One possible indirect pathogenesis pathway could be the anterograde and transsynaptic (transneuronal) degeneration of OB neural structures after damages or cell death of ORNs and olfactory nerve [120,121,122,123]. Moreover, even though axoplasmic transport of SARS-CoV-2 virus to the OB is rare, the situation seems different with regard to anterograde and transsynaptic transport of potentially pathogenic molecules and signals. SARS-CoV-2 spike protein cleavage peptide, for example, readily reaches the OB and other related CNS regions after intranasal instillation in the mice [73,124]. TLRs also participate in signaling between connected neurons in the olfactory system [119,125,126]. Finally, other CNS pathological changes, such as microvascular thrombosis, endothelium, and pericyte damages, microglia activation, and astrogliosis in the medulla oblongata, might have mainly or partially originated from the hematogenous route, and spread through the blood–brain barrier [74,75,83,92].

## 5. Persistent Anosmia, Hyposmia, or Parosmia

Most COVID-19 olfactory dysfunctions are transient, lasting for about 2–3 weeks. This is consistent with the fact that the OE undergoes regular ageing and self-replacement throughout life, and often readily repairs or regenerates itself upon damages [79,127,128,129]. In defiance of this well-known healing capability of the OE, however, a significant number of COVID-19 convalescents experience persistent ODs lasting for 12 months or longer [22,23,24,25]. The absent or exceptionally retarded recovery from COVID-19 ODs in those individuals implies a more severe or lasting damage to the OE by SARS-CoV-2. More specifically, this could result from SARS-CoV-2 infection or damage of the OE basal cells that express considerable ACE2 and TMPRSS2 [22,130]. Other possible causes of prolonged ODs after COVID-19 may pertain to persistent SARS-CoV-2 presence, chronic inflammation and immune reactions, or increased cell death in the OE. In COVID-19 convalescents with persistent anosmia, inflammation (as marked by infiltration of Iba1-positive myeloid cells), increased apoptosis (as marked by cleaved caspase 3-positive cells), and presence of SARS-CoV-2 (as marked by the viral nucleoprotein) could still be detected in the OE, but not in the RE, 6 months after the initial infection [23]. Interestingly, chronic inflammation could also modulate gene expression and switch the function of OE basal cells from neural regeneration to inflammatory signaling and immune cell proliferation [131].

## 6. Conclusions

SARS-CoV-2 has shown little neurotropism, apart from its high affinity to the neuron-supporting sustentacular cells of the OE. SARS-CoV-2 infection causes olfaction dysfunctions and ORN damages, most likely through indirect means such as deprivation of supports, inflammatory or immune reactions in the OE, and, to an extent, in the OB and other CNS regions (Figure 2B,C).

Apart from possible ORN anterograde degeneration and reactions affecting the OB and related CNS regions, SARS-CoV-2 infection and assaults on endothelial cells and pericytes of CNS vessels may cause microvascular thrombosis and leucocyte infiltration that, together with microglia activation, astrogliosis, inflammation, and immune responses, add to the pathogenic mechanisms for many of the COVID-19 neurological symptoms and complications (Figure 2D).

The causes and treatments of chronic SARS-CoV-2 infection of the OE and persistent post-COVID-19 ODs in a significant number of COVID-19 convalescents deserve further investigation.

## Data Availability

Not applicable.
